# Stress Relief Techniques: p38 MAPK Determines the Balance of Cell Cycle and Apoptosis Pathways

**DOI:** 10.3390/biom11101444

**Published:** 2021-10-02

**Authors:** Robert H. Whitaker, Jeanette Gowen Cook

**Affiliations:** Department of Biochemistry & Biophysics, University of North Carolina at Chapel Hill, Chapel Hill, NC 27599, USA; rhw@med.unc.edu

**Keywords:** cell cycle, cell signaling, apoptosis, mitosis, chemotherapy, kinase, protein networks, DNA damage, cell stress

## Abstract

Protein signaling networks are formed from diverse and inter-connected cell signaling pathways converging into webs of function and regulation. These signaling pathways both receive and conduct molecular messages, often by a series of post-translation modifications such as phosphorylation or through protein–protein interactions via intrinsic motifs. The mitogen activated protein kinases (MAPKs) are components of kinase cascades that transmit signals through phosphorylation. There are several MAPK subfamilies, and one subfamily is the stress-activated protein kinases, which in mammals is the p38 family. The p38 enzymes mediate a variety of cellular outcomes including DNA repair, cell survival/cell fate decisions, and cell cycle arrest. The cell cycle is itself a signaling system that precisely controls DNA replication, chromosome segregation, and cellular division. Another indispensable cell function influenced by the p38 stress response is programmed cell death (apoptosis). As the regulators of cell survival, the BCL2 family of proteins and their dynamics are exquisitely sensitive to cell stress. The BCL2 family forms a protein–protein interaction network divided into anti-apoptotic and pro-apoptotic members, and the balance of binding between these two sides determines cell survival. Here, we discuss the intersections among the p38 MAPK, cell cycle, and apoptosis signaling pathways.

## 1. Introduction

Cellular signal transduction pathways are crucial for the processes of development and differentiation and for homeostasis in adult tissues. Signaling pathways transmit information about both the intracellular and extracellular environment to elicit appropriate responses by changing gene expression, cell morphology, differentiation, cell proliferation, etc. For example, a specific tissue is maintained by a population of stem cells that remain dormant until extracellular signals in their environment indicate the need for proliferation. Those signals can be cell–cell contacts and/or growth factors that activate receptors, and the receptors trigger an intracellular cascade of protein interactions and reactions that ultimately induce cell division.

In addition to signals that induce proliferation or differentiation, there are signaling pathways that respond to stress. Broadly, cell stress can be categorized into both intracellular (replication stress, oxidation, etc.) and extracellular (radiation, UV, hypoxia, etc.). This categorization is not completely distinct because chemotherapy as an extracellular source of stress often results in DNA damage that can be thought of as an intracellular stress signal. At times the appropriate response to cell stress requires programmed cell death (apoptosis) to preserve the surrounding tissue. When cell stress signaling is dysregulated, the consequences can be inappropriate cell death (e.g., neurodegeneration) or inappropriate cell division (e.g., carcinogenesis).

Because cells experience multiple signals simultaneously, there is crosstalk between and among the signaling pathways, including the pathways controlling cell survival and proliferation. Although there are connections and intersections among many different signaling pathways and cell regulatory mechanisms, in this review we focus on the roles that the p38 stress pathway performs in the regulation of both cell cycle and cell survival pathways. p38 activation integrates and communicates stress signaling with survival and proliferation to accommodate stress. Here, we present the downstream impact of p38 signaling, specifically regarding apoptosis and cell cycle progression. Upstream signaling of p38, the differential roles of p38 isoforms, p38 activation and substrate recognition were recently reviewed in [1].

*MAP kinase signaling.* Mitogen Activated Protein Kinases (MAPKs) play central roles in multiple signaling pathways as intermediate transducers. MAPKs are converted from low activity to high activity states when they are phosphorylated by upstream protein kinases, most often one of the MEKKs (MAP and ERK kinases, also termed MAP2K). MEKs are themselves activated by upstream MEKK kinases (MEK kinases, or MAP3K) that are activated by many cytoplasmic factors that respond to a variety of upstream signaling events. The cascades of protein phosphorylation from MEKK to MEK to MAPK respond to different signals because the MEKKs and their activators are preferentially stimulated by different events. Alternate mechanisms of MAPK activation have also been reported (reviewed in [2,3]

The first human MAPK was originally identified as a phosphoprotein activated by several extracellular signals, such as insulin and growth factors, that can phosphorylate microtubule-associated protein-2 (MAP-2) in vitro [4,5]. The primary sequence of this enzyme, ERK1, was elucidated in 1990, and it showed strong similarity to two budding yeast kinases, Kss1 and Fus3 [6]. The Kss1 enzyme was discovered as a transducer of pheromone signaling in 1989 by Courschesne, Kunisawa, and Thorner, making it the founding member of the eukaryotic MAPK family [7]. Many family members were subsequently discovered in a wide range of eukaryotic species.

**Figure 1:**
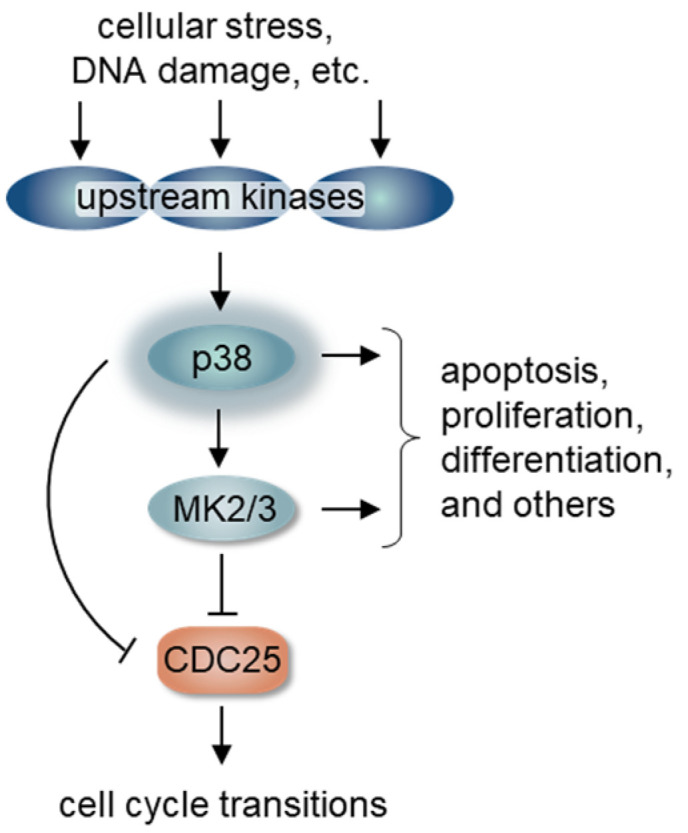
The stress activated p38 pathway. Cellular stress activates multiple upstream kinases such as MKK3/4. These upstream kinases phosphorylate and activate p38. Immediately downstream, p38 phosphorylates and activates MAPKAP-K2/3 (MK2/3). Both p38 and MK2/3 phosphorylate and inhibit the CDK-activating phosphatases, CDC25, to regulate cell cycle transitions.

MAPK enzymes are serine-threonine kinases that have strong substrate preference for either serine or threonine followed by a proline. This substrate preference is shared with a family of cell cycle regulatory kinases, the cyclin-dependent kinases (CDK), and MAPKs are evolutionarily most similar to CDKs [8]. MAPKs have been classified into 3 main subfamilies: ERK, JNK, and p38 based on sequence similarity and the specific signals that activate them. The ERK kinase subfamily (extracellular signal-regulated kinases) tend to be activated by extracellular mitogens and growth factors, whereas both c-Jun N-terminal kinases (JNK) and p38 MAP kinases respond robustly to both environmental stresses and intracellular signals [9]. For this reason, p38 signaling pathways have been canonically associated with both genotoxic stress and inflammation/immune responses; however, over the years the p38 signaling cascade has been demonstrated to also regulate differentiation, the cell cycle, and apoptosis (Figure 1) [10,11].

The p38 family consists of 4 isoforms: p38-α (also termed MAPK14), p38-β (MAPK11), p38-γ (MAPK12/ERK6), and p38-δ (MAPK13/SAPK4). Each isoform recognizes and phosphorylates the Ser-Pro or Thr-Pro dipeptide sequence found in consensus MAPK motifs. p38 is an intriguing but challenging drug target because it plays multiple and at times conflicting roles. Inhibiting p38 activity in vivo could have pleiotropic effects on many cellular pathways, some of which may be desirable and others problematic. Nonetheless, p38 inhibitors such as LY2282, LY3007113, and SCIO-469 have been developed and their efficacy alone or in combination with other therapies is being pursued in the treatment of cancer, inflammation, and neurodegeneration [1,12]. p38 inhibitors, including SB203580, have also been used extensively to assess the various roles of p38 in laboratory studies [13].

## 2. p38 Signaling and Cell Cycle Regulation

Cell stress can be anything that disrupts the homeostatic balance of normal cells, such as reactive oxygen species, bacterial toxins, changes in osmolarity, DNA damage, etc. Equipped with stress response pathways, including p38 signaling, cells adjust to these challenges to maintain cell function [14]. Once activated, the p38 signal cascade regulates the cell cycle by influencing both the timing of cell cycle entry and cell cycle checkpoint arrests that are necessary for cells to adjust to their environment. As part of the response to cell stress, p38 tends to act as a “brake,” inhibiting cell cycle transitions [15].

The cell cycle is a well-coordinated process necessary for accurate genome replication and segregation. This proliferative process includes a series of checkpoints that govern progression through the four primary cell cycle phases: G1 (gap 1), S (chromatin synthesis), G2 (gap 2), M (chromatin segregation and cell division). CDKs promote passage through these checkpoints and are activated by cyclin proteins whose abundance fluctuates in response to growth factor and mitogen signaling and throughout cell cycle phases [16]. The presence of cyclins (and other cell cycle factors) are also limited by ubiquitin-dependent proteolysis. Progression through G1 towards S phase (G1/S) is controlled by the CDK-RB-E2F pathway. RB, a major tumor suppressor and cell cycle inhibitor, is a pocket protein that binds and inhibits E2F transcription factors on chromatin [17,18].

**Figure 2:**
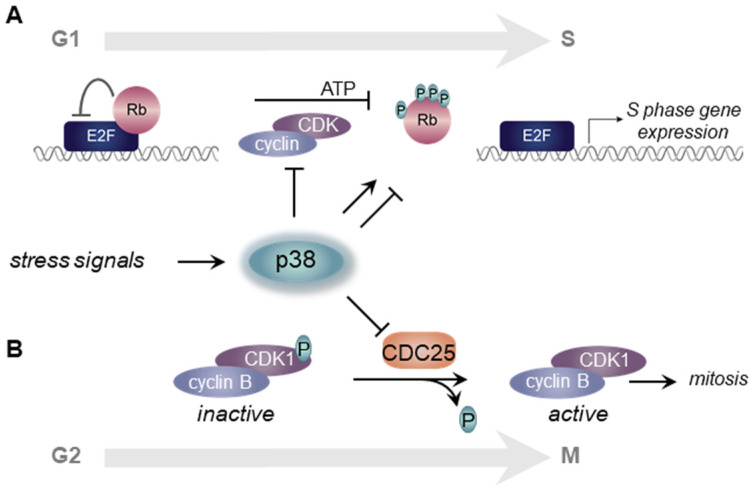
p38 regulates G1/S and G2/M cell cycle transitions. (**A**) The G1/S transition is controlled by Rb phosphorylation and CDK status. When activated by cellular stress, p38α phosphorylation of RB maintains inhibition of E2F. p38 also mediates G1 arrest by phosphorylating cyclin D resulting in its degradation. Alternatively, p38γ phosphorylates Rb similar to CDK releasing E2F inhibition. (**B**) The G2 transition into mitosis is controlled by cyclin B-activated CDK1. During G2, phospho-CDK1 is inactive, and the dephosphorylation of CDK1 by CDC25 activates CDK1/cyclin B. In response to cell stress, active p38 (and p38-activated MK2) inhibit CDC25 arresting cells in G2.

*p38 influences G1 progression.* The E2F family of transcription factors activates genes necessary for DNA replication during S phase as well as genes encoding cyclins that act at later cell cycle phases. The RB protein inhibits E2F on chromatin when in its hypophosphorylated state, thereby preventing transcriptional upregulation of the genes necessary for entry and completion of S phase [19]. The G1 CDKs hyperphosphorylate RB to induce E2F release and subsequent S phase gene activation (Figure 2A) [20,21]. Mitogens stimulate cyclin D expression during G1 through activation of the ERK MAPK pathway [22,23]. Cyclin D binds and activates CDK4 and/or CDK6 to monophosphorylate RB at multiple different sites. Although the mechanisms are still not fully understood, at least some forms of monophosphorylated RB permit E2F de-repression and cyclin E induction later in G1 [24,25,26]. In late G1, cyclin E binds and activates CDK2 to hyper-phosphorylate RB causing complete release of E2F which then strongly stimulates the transcriptional program to initiate DNA replication and S phase [27,28]. CDKs are themselves controlled by multiple molecular mechanisms including critical contributions from CDK inhibitor proteins. These inhibitors include the CIP/KIP family (p21, p27, p57) that inhibits CDK2 and the INK4 family (p15, p16, p18, p19) that inhibits CDK4 and CDK6 [29,30].

During G1 phase, p38 can play dual roles depending on context and cell type. Most often, p38 inhibits G1 progression, meaning that p38 kinase activity must be low to move past G1 and begin DNA replication in S phase [15,31]. If p38 activity is high in response to a stress signal then the G1/S transition is inhibited. The specific mechanism of p38-mediated G1 inhibition includes phosphorylation of several proteins involved in the CDK-RB-E2F pathway. For example, active p38α phosphorylates cyclin D (Thr 286) resulting in this cyclin’s ubiquitination and degradation [32]. p38 also phosphorylates and stabilizes the CDK inhibitor p21 and induces p16 [33,34,35]. Interestingly, p38 can phosphorylate RB in a manner independent of CDK, but unlike CDK-mediated phosphorylation, p38 phosphorylates RB at distinct sites that strengthen E2F repression [36]. In this case, p38 phosphorylates RB on its N-terminal domain, distant from the C-terminal pocket domain where CDK phosphorylates RB to release E2F. This allosteric-type p38-mediated phosphorylation of RB prevents release and activation of E2F. Of note however, and unlike p38α, the gamma isoform of p38 can phosphorylate RB in a manner reminiscent of CDK that releases E2F to promote entry into S phase [37].

*p38 influences G2 progression*. Stress-activated p38 also prevents the G2 to M phase cell cycle transition through checkpoint mechanisms in G2 phase. The G2/M checkpoint is a complex regulatory checkpoint that ensures that DNA damage or defects during DNA replication are rectified before initiating mitosis. Many forms of DNA damage activate p38, and the sources of that damage can be external, such as from UV irradiation [38,39], or generated endogenously from replication fork stalling [40], ribosome stalling [41], or even the double-strand breaks created for VD (J) recombination during T cell development [42]. The principal target of this checkpoint is the activity of Cyclin B-activated CDK1 which is crucial for transition into and progression through mitosis. High p38 activity can prevent G2 progression to mitosis by preventing the activation of cyclin B/CDK1 [43].

One of the primary substrates of p38 is the MAPKAPK2 (MAPK-activated protein kinase 2 or MK2) which is directly activated by p38-mediated phosphorylation. MK2 phosphorylates the CDC25B and CDC25C phosphatases which are essential to remove inactivating phosphates from CDK1 (Figure 1) [38]. Phosphorylation of CDC25B/C by MK2 induces its sequestration and cytoplasmic retention [38]. p38 can also directly phosphorylate CDC25B/C to induce degradation or relocalization [44,45]. Taken together, high p38 activity arrests cells in G2 through CDC25B/C inhibition and consequent failure to activate cyclin B/CDK1 (Figure 2B) [44,46]. The p38/MK2 pathway also promotes prolonged G2 arrest by reinforcing CDC25B/C cytoplasmic retention, and this function may be particularly important in cells that have lost the p53 tumor suppressor [47]. Interestingly, after mitotic entry, p38 is once again required during mitosis for the stable attachment of kinetochores to spindle microtubules, and in the absence of p38 these spindles were significantly longer. The formation of these longer spindles delayed passage through the spindle assembly checkpoint [48].

Aside from directly engaging cell cycle progression mechanisms, p38 also acts as a mediator of cell survival during the cell cycle. For example, inactivating p38 during G2 can trigger p53-independent apoptosis [34,49]. These findings highlight the importance of the balance between cell cycle progression and apoptosis during times of cellular stress or damage. We next turn our attention to connections between the pathways that control cell death by apoptosis and core cell cycle pathways. We focus on connections between the cell cycle and the BCL2 family of cell survival regulators.

## 3. The BCL2 Family and the Cell Cycle

The BCL2 family are key regulators of cell survival and the penultimate step in stress signaling before caspase activation to induce cell death by apoptosis (i.e., programmed cell death). Cellular life is maintained by a balance between survival and proliferative signaling networks. Both networks involve competition between positive and negative regulators. When the balance is disrupted, cells may display aberrant survival and sustained proliferative signaling, two of the hallmarks of cancer [50]. The BCL2 family dictates survival through the relative concentrations and binding between its pro- and anti- apoptotic members [51]. Anti-apoptotic and pro-apoptotic proteins form a homeostatic balance that can tip a cell towards survival or programmed cell death depending on the overall stress level of that cell.

**Figure 3:**
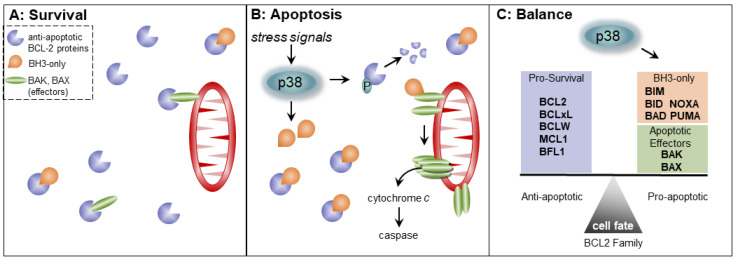
p38 stress signaling tips the balance of BCL2 family interactions towards cell death. (**A**) Pro-survival proteins (BCL2, MCL1, etc.) mediate survival by binding and preventing apoptotic effectors (BAK and BAX) from pores in the outer mitochondrial membrane. The anti-apoptotic proteins bind and inhibit both the pro-apoptotic BH3-only proteins and apoptotic effectors. (**B**) During cell stress, active p38 upregulates expression of BH3-only proteins (BIM, BID, etc.). These BH3-only proteins can either directly activate BAK or BAX or inhibit anti-apoptotic proteins. Active p38 can phosphorylate and induce degradation of some anti-apoptotic proteins. These stress-initiated events lead to the homo-oligomerization of the BCL2 effectors causing membrane permeabilization, cytochrome c release, caspase activation, and cell death. (**C**) Stress-activated p38 signaling shifts the balance of the BCL2 family towards apoptosis.

*Interactions and functions of BCL2 proteins*. Broadly, BCL2 proteins can be divided into both pro- and anti-apoptotic factors (Figure 3A). Intracellular stress signals and overall cell health control the ratio of the two types. Cell survival is promoted when the anti-apoptotic proteins bind and keep the pro-apoptotic proteins in check [52,53]. These anti-apoptotic proteins are globular, folded proteins formed by a series of BCL2 Homology domains (BH) [54,55,56]. The BH helices form a common hydrophobic groove or cleft, able to bind an individual small protein domain, known as a BH3 motif [57]. Although it is found in all BCL2 members, the BH3 motif is exposed in the pro-apoptotic proteins and readily available for interaction with and inhibition by the anti-apoptotic proteins. These internal family interactions and dynamics are reviewed in [58]. The anti-apoptotic proteins include BCL2, BCLxL, MCL1, BCLW, and BFL1; while the pro-apoptotic proteins branch into two groups: the BCL2 effectors and BH3-only proteins (including BIM, BID, BAD, etc.) (Table 1) [52,58].

BAK and BAX are pro-apoptotic effectors that directly induce apoptosis through mitochondrial damage if they are not bound by the anti-apoptotic members. Left unchecked, these BCL2 apoptotic effectors homo-oligomerize and permeabilize the outer mitochondrial membrane. Mitochondrial outer membrane permeabilization (MOMP) releases Cytochrome C and results in caspase activation and cell death. The BH3-only proteins are upstream of these events and interact with both the pro-survival proteins and pro-apoptotic effectors. Stress signaling (genotoxic, chemotoxic, etc.) upregulates expression of the BH3-only proteins [52,59,60]. Under these conditions, the increasing ratio of BH3-only members outcompetes BAK/BAX interaction with the anti-apoptotic proteins, freeing the effectors to cause MOMP (Figure 3B). Additionally, some BH3-only proteins can stimulate apoptosis more directly by activating BAK/BAX for polymerization.

As key regulators of cell death, the hydrophobic binding pocket of anti-apoptotic BCL2 proteins has been a target of chemotherapy development [61]. As the natural binding partner of this pocket, the BH3 motif has been used in drug discovery to identify other compounds that bind and inhibit the anti-apoptotic function of these proteins [52]. Using these compounds perturbs the BCL2 family dynamics by inhibiting the anti-apoptotic members and freeing the apoptotic effectors to act. These drugs show promise both as solo therapies or combined with established ones [62]. So far, one BCL2 inhibitor, venetoclax, has been approved to treat chronic lymphocytic leukemia [63,64].

*Anti-apoptotic BCL2 family proteins influence the cell cycle.* Since the discovery of the BCL2 family as critical regulators of survival, additional roles for this family have been hypothesized including participating in proliferative signaling and cell cycle regulation [65]. There is increasing evidence that the BCL2 family directly interacts with the cell cycle machinery. Individual anti-apoptotic members have been detected in different cell cycle phases, so one can imagine specific BCL2 proteins inhibiting apoptosis during specific phases of the cell cycle—a relay race of survival signaling.

Beginning in G1 the anti-apoptotic proteins, BCL2 and BCLxL, maintain cellular viability and prolong G1. One role of BCL2 during G1 is to induce the CDK inhibitor, p27, by a mechanism not yet fully understood [66,67,68,69]. An interesting proposed mechanism relies on BCL2-dependent suppression of reactive oxygen species (ROS) that in turn, downregulate p27. Phosphorylated BCL2 (Thr69/Ser70/Ser87) acts as an antioxidant to reduce the abundance of ROS. [70].

Interestingly, the role of BCL2 during G1 appears to be different from another anti-apoptotic protein, MCL1. Natural Killer (NK) cells have higher amounts of BCL2 in non-cycling cells, while MCL1 was more abundant in cycling NK cells [71]. Our own work (RHW), has detailed a mechanism for MCL1 to promote the G1/S transition by binding and destabilizing the early G1 cell cycle inhibitor, p18 (CDKN2C, P18INK4C) that inhibits CDK4/6 kinases, thereby pushing cells into S phase [72]. Specifically, the MCL1 binding pocket interacts with a BH3-like motif in p18, the “reverse BH3” [73]. Thus, MCL1-mediated p18 destabilization primes entry into S phase.

In S phase, MCL1 may interact directly with PCNA, a processivity factor for DNA polymerase needed during DNA synthesis [74]. A canonical PCNA-binding sequence motif in MCL1 interacts with PCNA, and overexpression of MCL1 decreased S phase and replication as shown by BrdU uptake [74]. Like p38, MCL1 appears to lie at a nexus where this single protein can interpret a variety of signals from the cellular milieu at the G1/S transition and thereby direct cells towards different fates.

During mitosis, the abundance of MCL1 acts as a mitotic timer. MCL1 has a short half-life because it is targeted for degradation by at least three different ubiquitin ligases (Mule/HUWE1, SCF, and APC/C [75,76,77,78]). Targeting by the Anaphase Promoting Complex/Cyclosome (APC/C) occurs by an atypical mechanism activated by cyclin B/CDK1 that results in a steady MCL1 decline rather than an abrupt loss at anaphase [79]. In a normal mitosis, MCL1 sustains cell survival, and as mitosis completes, MCL1 levels stabilize to promote survival because CDK1 activity decreases after anaphase [77,79]. Upon prolonged mitotic arrest, MCL1 levels continue to decrease throughout the arrest until MCL1 levels are below a threshold and can no longer perform the anti-apoptotic role [79]. This pattern connects to the general anti-apoptotic role of MCL1; levels that decrease below a certain threshold for restraining proapoptotic BCL2 family members allow a mitotically paused cell to apoptose [80]. Neuronal cells exiting a final mitosis also rely on MCL1 not only for survival but also to promote terminal differentiation through upregulation of the p27 CDK inhibitor [81].

*Pro-apoptotic BCL2 family proteins influence the cell cycle.* The pro-apoptotic side of the BCL2 family also interlinks with the cycle. During quiescence (also termed G0), the phosphorylation status of the pro-apoptotic and BH3-only protein, BID, dictates its role in cell cycle progression. When phosphorylated by ATM in response to DNA damage (at sites S61/S78), BID maintains hematopoietic stem cells (HSC) by both sustaining viability and preventing ROS stimulated entry into S phase [82]. Presumably, this protection occurs through the association of phosphorylated BID with MTCH2, a negative regulator of mitochondrial oxidative phosphorylation [83]. Increasing levels of oxidative stress result in loss of BID phosphorylation and upregulation of the *cyclin D*, *cyclin E* and *CDK4* genes. Expression of these genes supports HSC self-renewal, though the precise mechanisms regulating redox signaling and cell fate have not been fully worked out [83]. However, if the oxidative stress becomes too great, BID is dephosphorylated and performs its canonical pro-apoptotic function.

Of note, mitotic BID phosphorylation at S66, a site different from the ATM targets, promotes BID’s pro-apoptotic function [84]. BID participation spans mitosis, cell cycle exit into G0, and re-entry back into G1 [83,85,86]. Following mitosis, the key G1 phase transcription factor, E2F, upregulates several other BH3-only pro-apoptotic proteins, including BIM, NOXA, and PUMA [87]. Somewhat surprisingly BAX (a MOMP effector) enhances S phase entry measured by BrdU uptake rather than inducing apoptosis, and correlates with degradation of the cell cycle inhibitor p27 [88,89,90]. This increase in proliferative potential appears to contradict BAX’s pro-apoptotic function and suggests context-specific roles for BAX.

*The cell cycle influences BCL2 family proteins.* The connections between the cell cycle and the BCL2 family are not unidirectional since cell cycle regulators also impact BCL2 proteins. RB, a major tumor suppressor whose ability to inhibit E2F is altered in most if not all cancers, also regulates apoptosis [91,92]. E2F, the central G1-S transcription factor, directly suppresses the promoters of anti-apoptotic BCL2 and MCL1 [93,94]. Strikingly, RB not only functions as a transcriptional repressor in the nucleus, but it is also found at mitochondria. RB at the mitochondrial outer membrane associates with the pro-apoptotic BCL2 effector, BAX [95,96]. However, the phosphorylation status of RB determines whether its interaction with BAX is pro-apoptotic or anti-apoptotic. The internal portion (residues 373–766) of phospho-RB (Ser608, Ser708) can interact and directly activate BAX (but not BAK) resulting in apoptosis [95]. However, phosphorylated RB (Ser608, Ser795, Ser807/Ser811, and Thr821) binds and inhibits BAX until RB is dephosphorylated [96]. Although these opposing outcomes were elucidated in different cell lines (normal mouse fibroblasts vs. human breast cancer cells), the location of phosphorylated residues on RB appears to define its involvement in survival.

The at-times contradictory conclusions about BCL2 member activities throughout the cell cycle show the need for working out the specific mechanisms that connect the cell cycle with BCL2-mediated survival. It is anticipated that more non-canonical roles beyond regulating apoptosis for BCL2 proteins will be discovered, including more links to the cell cycle.

## 4. p38 MAPK Signaling and Regulation of Apoptosis by the BCL2 Family

Connections between apoptosis and all three MAPK pathways have been reviewed in [97,98]. p38 pathway activation is one route upstream of caspase activation to induce apoptosis. Although internal BCL2 family binding dynamics are based on hydrophobic pockets and amphipathic helices, individual BCL2 proteins are also regulated by post-translational modifications, namely phosphorylation, that typically impact BCL2 stability. For example, both BCL2 and MCL1 are phosphorylated by CDK1 resulting in their ubiquitination and degradation [99]. These post-translational modifications allow kinase pathways like p38 to impact the stability and therefore availability of BCL2 members to perform either their pro- or anti- apoptotic functions.

Specifically, p38 phosphorylates several anti-apoptotic BCL2 proteins, including BCL2, MCL1, and BCLxL leading to their ubiquitination and degradation which tips the scales towards cell death [77,100,101,102]. However, p38 upregulates MCL1 transcription in the context of prostate cancer which may counteract p38-induced degradation [103,104]. In particular, cells are incredibly sensitive to the concentration of MCL1 which has a very short half-life of ~30 min, and it must be continually either expressed or stabilized to prevent MCL1 levels from dropping [105,106]. Both cell type and environment are relevant for how cells resolve these conflicting p38 effects. Keeping cells poised between the two fates of cell death and life promotes rapid responses to environmental changes.

Beyond phosphorylation, p38 also targets BCL2 and MCL1 expression transcriptionally [100,103]. In the case of BCL2, p38 downregulates BCL2 transcription which prevents neuronal differentiation [100]. For decades, BCL2 has been noted for its role in neuronal differentiation, where BCL2 overexpression increased neurite formation without impacting cell survival [107]. In contrast to BCL2, the anti-apoptotic MCL1 has been inversely correlated with differentiation in both HSCs and neuronal cells [55,108]. p38 itself also plays a variety of roles in lineage-specific differentiation (reviewed in [109])

The p38 pathway regulates of pro-apoptotic BCL2 proteins, including BCL2 apoptotic effectors and the BH3-only proteins. The effector BAX is phosphorylated by p38 which protects it from binding anti-apoptotic BCL2 and promotes apoptosis [110]. Cell stress induces expression of BCL2 pro-apoptotic BH3-only members priming the apoptotic response. Oxidative stress activates p38 to phosphorylate BIM (Ser65). This phosphorylated BIM more robustly induces apoptosis than unphosphorylated BIM [111]. Additionally, active p38 induces transcription of the BIM gene thereby increasing cell death [112]. This p38-dependent expression of BIM appears to be dependent on the forkhead transcription factor FOXO3a [113]. Another BH3-only pro-apoptotic protein, PUMA, is transcriptionally activated by p38 in a p53-dependent manner [39,114]. Additionally, a different BH3-only protein, NOXA, is transcriptionally upregulated in response to ROS by p38 [115]. Moreover, p38 has been implicated in an undefined mechanism in the translocation of PUMA to the mitochondria where it can promote apoptosis [116,117]. In a reverse of the relationship, one report indicates that the BH3-only NOXA localized at the endoplasmic reticulum can promote p38 activation by ROS through a possible mechanism involving the apoptosis signal-regulating kinase (ASK1) [118].

Two specific cellular contexts highlight the interplay between the BCL2 family and p38—cardiac cells and neurons. While an imbalance of the BCL2 family in favor of anti-apoptotic members is often carcinogenic, tipping the BCL2 family ratio towards the pro-apoptotic can result in neurodegeneration or cardiac hypertrophy/cell death depending on cell lineage and p38 isoform. In the brain, p38 increases expression of pro-apoptotic BAX resulting in neurodegeneration through increased cell death [119]. As referenced earlier, p38 and BCL2 participate in neuronal commitment and differentiation where BCL2 maintains cell viability [100]. More recently, the presence of BCL2 increased the conversion of cells into neurons by direct reprogramming [120]. Interestingly, this glial-to-neural differentiation was independent of the anti-apoptotic function of BCL2 as shown through inactivating point mutations [120].

In cardiac tissue, chemical inhibition of p38 (but not ERK) downregulated BCL2 in the context of hypoxia [121]. Further, this downregulation of BCL2 limited vascular endothelial cell proliferation possibly through decreased microtubule formation [121]. Intriguingly, these models of hypoxia suggest the involvement of both p38 and BCL2 signaling during quiescence similar to their effects in quiescent cultured cells [90,122]. The specific mechanism of p38 effects on BCL-2 family expression still needs to be fully elucidated.

## 5. Discussion

In the context of cancer, p38 was originally identified as a tumor suppressor, but it became apparent that its role is more complicated since it can be both tumor promoting and suppressing. To disentangle these disparate observations regarding p38 in tumorigenesis, it has been suggested that p38 is tumor suppressing early in tumorigenesis but after cellular transformation, p38 activity becomes tumor promoting [1,123]. Here, we suggest that the actions of p38 at different points within the cell cycle contribute to the dual function of p38 signaling. This duality is clearly evidenced by the variable impact of p38 targeting for therapy.

The relationship between p38 and cancer therapy is tangled because it is often through p38 that chemotherapy induces cell death, whereas in other scenarios, p38 acts like an oncogene [1,123,124]. Attempting to target p38 alongside traditional therapies like microtubule targeting agents (MTAs) is a common strategy, but the results are context dependent. Indeed, early reports utilized spindle poisons like nocodazole to study p38 activation [125]. MTAs, including taxanes and vinca alkaloids, are standard cytotoxic chemotherapies that inhibit microtubule dynamics during mitosis causing mitotic arrest and cell death [62]. p38 mediates taxane-induced cell death in HeLa cells and breast cancer patient-derived xenografts [126,127]. However, in other contexts like gastric and prostate cancer, increased p38 activity is associated with drug resistance [128,129]. Other genotoxic therapies, including cisplatin and doxorubicin, also require the influence of active p38 on the DNA damage response to mediate apoptosis [130,131].

p38 participation in the G2/M checkpoint has been expanded to connect to cell survival maintained by the BCL2 family [34,49]. It has also been noted that when p38 is decreased, so is MCL1 [123]. The crosstalk between p38 MAPK signaling and the BCL2 family, necessitates the consideration of both when either pathway is targeted. Surprisingly, little is known about the outcome of combining both anti-apoptotic BCL2 family, and p38 pathway inhibitors although both are critical in the response to cell stress. Combination therapy is foundational to cancer treatment where targeting inter-connected pathways may result in synergy and decreased drug resistance. The timing of such a targeting arrangement would need to be precise due to the potential for p38 to act as both a tumor promoter and suppressor. Specifically, the influence of p38 on the BCL2 family in different types of cancer should be evaluated carefully as the field moves towards precision medicine. As a therapeutic example, p38 inhibition could lead to less pro-survival MCL1 through increased CDK1 activation. Here, it might be beneficial to pair p38 inhibition with an inhibitor targeting a different BCL2 member like BCL2 itself because low MCL1 plus inactivated BCL2 strongly favor apoptosis.

To add more complexity to these considerations, p38 influences cell death mechanisms that are independent of controlling BCL2 family members. One parallel apoptotic pathway is downstream of tumor necrosis factor, an extracellular inflammatory cytokine that activates p38. The MK2 kinase that is then activated by p38 is induced to phosphorylate and inhibit the RIPK1 kinase, a caspase activator [132,133,134]. In this context, p38 signaling restrains apoptosis.

The difficulty in pairing p38 inhibitors with other chemotherapies stems from the opposing roles p38 plays in stress responses during disease development and progression. p38 is poised to allow cells to adapt and deal with a variety of stressors. For instance, in colon cancer p38 protects against transformation of normal cells, but once transformed, colorectal cancer cells need p38 for proliferation and survival as shown by tissue-specific knockout experiments [123]. Mechanistically, this difference may be attributed to the degree of p38 activation in which low p38 activation promotes survival but robust and prolonged p38 activation promotes alternative cell fates including death or senescence [135,136]. This dual role of p38 has been attributed to several potential causes including the changing external environment from initiation to cancer transformation, transient p38 activation versus persistent activation, or the type of p38 activating signal (mitogen versus chemotherapy) [137]. This places p38 as a rheostat whose dial is controlled by the level of p38 activation, type of signal input, and the surrounding cellular environment.

It also seems that these disparate mechanisms resolve when seen through the lens of the cell cycle. We suggest that the cell cycle phase when p38 is activated or targeted is critical in determining its role in both disease development and therapy response. Specifically, mitogen signaling, transient activation, and suppressing cancer initiation appear to fit into the role of p38 during the G1/S transition. On the other hand, persistent activation, chemotherapeutic activation, and facilitating cancer progression are linked with the role of p38 in G2/M and survival during a G2 arrest. In this model, normal cycling cells with transient p38 activation would pass the G1 checkpoint before DNA replication in S phase. Naturally, this kind of p38 activation would be induced by mitogens and growth factors. Alternatively, the conditions of replication stress and polyploidy during cancer progression or increasing chemotherapy exposure with subsequent DNA damage would induce persistent p38 activation.

Does p38 play a role in BCL2-maintained survival at the G1/S transition like it does during G2/M? It is vital to understand how p38 signaling influences cell survival and the BCL2 family at the G1/S transition, as the ambiguity of a p38-induced arrest during cancer treatment may result in repair, senescence, or death. In the case of a prolonged arrest during G1, p38 inhibition might permit more cells to progress into S, G2, and M phases and therefore become vulnerable to cytotoxic therapies that target DNA replication or mitosis.

Factors like p38 and MCL1 not only transmit cell signals but also modulate incoming molecular messages to direct cell fate. While challenging to assess, the nuances of these biological outcomes appear in the contradictory yet empirical observations reported in the literature. These contradictions raise important questions, beyond the specificity of different cell lineages and cancer types: where and when is the p38 “rheostat” a main player? Does p38 only connect to cell cycle and survival regulation under conditions of stress or does it govern cell cycle progression under normal conditions? Answering questions like these will not only determine the precise mechanism of cancer development but when and where to employ inhibitors of these protein networks during disease progression.

## Figures and Tables

**Table 1:** List of key proteins discussed in the text (relevant references in the text).

Protein Family/Category	Protein	Cell Cycle and Survival Roles	p38 Integration/Effect
BCL2 Anti-Apoptotic Proteins	BCL2	G1 phase ↑; survival	+P degradation; transcription **↓**
	MCL1	G1/S trans. ↑, M phase timer; survival	+P degradation; transcription **↓**
	BCLxL	G1 phase ↑; survival	+P degradation
BCL2 Apoptotic Effectors	BAX	RB interaction; apoptosis	+P apoptosis ↑
	BAK	S phase ↑; apoptosis	-
BCL2 BH3-Only Proteins	BIM	transcription ↑ by E2F; apoptosis	+P, apoptosis ↑; transcription ↑
	BID	G0/G1, M; apoptosis	-
	NOXA	transcription ↑ by E2F; apoptosis	transcription ↑
	PUMA	transcription ↑ by E2F; apoptosis	transcription ↑
	cyclin D	CDK4/6 activation	+P degradation
	CDK4/6	RB inhibition	inhibits
Cell Cycle: G1/S Transition	RB	E2F inhibition; (BAX interaction)	p38α activates RB; γ inhibits
	E2F	S transcription; (BCL2/MCL1 transcription **↓**)	-
	cyclin E	CDK2 activation	-
	CDC25	CDK dephosphorylation and activation	inhibition
	cyclin B	CDK1 activation	-
Cell Cycle: G2/M Transition	CDK1	M entry and progression	-
	CDC25	CDK dephosphorylation and activation	inhibition
	p27	CDK2 inhibitor; (MCL1 & terminal mitosis)	
Cell Cycle Inhibitors	p18	CDK4/6 inhibitor; (destabilized by MCL1)	-
	p38	Cell cycle arrest in response to stress	-
p38 MAPK Pathway	MK2	CDC25 inhibition and cycle arrest	+P activated

“+P” indicates the effect of p38-mediated phosphorylation. ↑ and ↓ indicate induction and repression, respectively.

## Data Availability

Not applicable.

## References

[1] Martinez-Limon A., Joaquin M., Caballero M., Posas F., de Nadal E. (2020). The p38 Pathway: From Biology to Cancer Therapy. Int. J. Mol. Sci.

[2] Dhanasekaran D.N., Johnson G.L. (2007). MAPKs: Function, regulation, role in cancer and therapeutic targeting. Oncogene.

[3] Canovas B., Nebreda A.R. (2021). Diversity and versatility of p38 kinase signalling in health and disease. Nat. Rev. Mol. Cell Biol..

[4] Ray L.B., Sturgill T.W. (1987). Rapid stimulation by insulin of a serine/threonine kinase in 3T3-L1 adipocytes that phosphorylates microtubule-associated protein 2 in vitro. Proc. Natl. Acad. Sci. USA.

[5] Hoshi M., Nishida E., Sakai H. (1988). Activation of a Ca2+-inhibitable protein kinase that phosphorylates microtubule-associated protein 2 in vitro by growth factors, phorbol esters, and serum in quiescent cultured human fibroblasts. J. Biol. Chem..

[6] Boulton T.G., Yancopoulos G.D., Gregory J.S., Slaughter C., Moomaw C., Hsu J., Cobb M.H. (1990). An insulin-stimulated protein kinase similar to yeast kinases involved in cell cycle control. Science.

[7] Courchesne W.E., Kunisawa R., Thorner J. (1989). A putative protein kinase overcomes pheromone-induced arrest of cell cycling in *S. cerevisiae*. Cell.

[8] Manning G., Whyte D.B., Martinez R., Hunter T., Sudarsanam S. (2002). The protein kinase complement of the human genome. Science.

[9] Westfall P.J., Ballon D.R., Thorner J. (2004). When the stress of your environment makes you go HOG wild. Science.

[10] Nebreda A.R., Porras A. (2000). p38 MAP kinases: Beyond the stress response. Trends Biochem. Sci..

[11] Wagner E.F., Nebreda A.R. (2009). Signal integration by JNK and p38 MAPK pathways in cancer development. Nat. Rev. Cancer.

[12] Cohen P. (2009). Targeting protein kinases for the development of anti-inflammatory drugs. Curr. Opin. Cell Biol..

[13] Cuenda A., Rousseau S. (2007). p38 MAP-kinases pathway regulation, function and role in human diseases. Biochim. Biophys. Acta.

[14] Lu S., Wei F., Li G. (2021). The evolution of the concept of stress and the framework of the stress system. Cell Stress.

[15] Thornton T.M., Rincon M. (2009). Non-classical p38 map kinase functions: Cell cycle checkpoints and survival. Int. J. Biol. Sci..

[16] Evans T., Rosenthal E.T., Youngblom J., Distel D., Hunt T. (1983). Cyclin: A protein specified by maternal mRNA in sea urchin eggs that is destroyed at each cleavage division. Cell.

[17] Friend S.H., Bernards R., Rogelj S., Weinberg R.A., Rapaport J.M., Albert D.M., Dryja T.P. (1986). A human DNA segment with properties of the gene that predisposes to retinoblastoma and osteosarcoma. Nature.

[18] Chellappan S.P., Hiebert S., Mudryj M., Horowitz J.M., Nevins J.R. (1991). The E2F transcription factor is a cellular target for the RB protein. Cell.

[19] Frolov M.V., Dyson N.J. (2004). Molecular mechanisms of E2F-dependent activation and pRB-mediated repression. J. Cell Sci..

[20] Ubersax J.A., Ferrell J.E. (2007). Mechanisms of specificity in protein phosphorylation. Nat. Rev. Mol. Cell Biol..

[21] Brown V.D., Phillips R.A., Gallie B.L. (1999). Cumulative effect of phosphorylation of pRB on regulation of E2F activity. Mol. Cell Biol..

[22] Albanese C., Johnson J., Watanabe G., Eklund N., Vu D., Arnold A., Pestell R.G. (1995). Transforming p21 Mutants and c-Ets-2 Activate the Cyclin D1 Promoter through Distinguishable Regions. J. Biol. Chem..

[23] Duronio R.J., Xiong Y. (2013). Signaling Pathways that Control Cell Proliferation. Cold Spring Harb. Perspect. Biol..

[24] Narasimha A.M., Kaulich M., Shapiro G.S., Choi Y.J., Sicinski P., Dowdy S.F. (2014). Cyclin D activates the Rb tumor suppressor by mono-phosphorylation. eLife.

[25] Sanidas I., Morris R., Fella K.A., Rumde P.H., Boukhali M., Tai E.C., Ting D.T., Lawrence M.S., Haas W., Dyson N.J. (2019). A Code of Mono-phosphorylation Modulates the Function of RB. Mol. Cell.

[26] Zarkowska T., Mittnacht S. (1997). Differential phosphorylation of the retinoblastoma protein by G1/S cyclin-dependent kinases. J. Biol. Chem..

[27] Johnson D.G., Ohtani K., Nevins J.R. (1994). Autoregulatory control of E2F1 expression in response to positive and negative regulators of cell cycle progression. Genes Dev..

[28] Slansky J.E., Farnham P.J. (1996). Introduction to the E2F family: Protein structure and gene regulation. Curr. Top. Microbiol. Immunol..

[29] Besson A., Dowdy S.F., Roberts J.M. (2008). CDK inhibitors: Cell cycle regulators and beyond. Dev. Cell.

[30] Canepa E.T., Scassa M.E., Ceruti J.M., Marazita M.C., Carcagno A.L., Sirkin P.F., Ogara M.F. (2007). INK4 proteins, a family of mammalian CDK inhibitors with novel biological functions. IUBMB Life.

[31] Kishi H., Nakagawa K., Matsumoto M., Suga M., Ando M., Taya Y., Yamaizumi M. (2001). Osmotic shock induces G1 arrest through p53 phosphorylation at Ser33 by activated p38MAPK without phosphorylation at Ser15 and Ser20. J. Biol. Chem..

[32] Casanovas O., Miro F., Estanyol J.M., Itarte E., Agell N., Bachs O. (2000). Osmotic stress regulates the stability of cyclin D1 in a p38SAPK2-dependent manner. J. Biol. Chem..

[33] Faust D., Dolado I., Cuadrado A., Oesch F., Weiss C., Nebreda A.R., Dietrich C. (2005). p38alpha MAPK is required for contact inhibition. Oncogene.

[34] Bulavin D.V., Higashimoto Y., Popoff I.J., Gaarde W.A., Basrur V., Potapova O., Appella E., Fornace A.J. (2001). Initiation of a G2/M checkpoint after ultraviolet radiation requires p38 kinase. Nature.

[35] Kim G.Y., Mercer S.E., Ewton D.Z., Yan Z., Jin K., Friedman E. (2002). The stress-activated protein kinases p38 alpha and JNK1 stabilize p21(Cip1) by phosphorylation. J. Biol. Chem..

[36] Gubern A., Joaquin M., Marques M., Maseres P., Garcia-Garcia J., Amat R., Gonzalez-Nunez D., Oliva B., Real F.X., de Nadal E. (2016). The N-Terminal Phosphorylation of RB by p38 Bypasses Its Inactivation by CDKs and Prevents Proliferation in Cancer Cells. Mol. Cell.

[37] Tomas-Loba A., Manieri E., Gonzalez-Teran B., Mora A., Leiva-Vega L., Santamans A.M., Romero-Becerra R., Rodriguez E., Pintor-Chocano A., Feixas F. (2019). p38gamma is essential for cell cycle progression and liver tumorigenesis. Nature.

[38] Manke I.A., Nguyen A., Lim D., Stewart M.Q., Elia A.E., Yaffe M.B. (2005). MAPKAP kinase-2 is a cell cycle checkpoint kinase that regulates the G2/M transition and S phase progression in response to UV irradiation. Mol. Cell.

[39] Bulavin D.V., Saito S., Hollander M.C., Sakaguchi K., Anderson C.W., Appella E., Fornace A.J. (1999). Phosphorylation of human p53 by p38 kinase coordinates N-terminal phosphorylation and apoptosis in response to UV radiation. EMBO J..

[40] Kopper F., Bierwirth C., Schon M., Kunze M., Elvers I., Kranz D., Saini P., Menon M.B., Walter D., Sorensen C.S. (2013). Damage-induced DNA replication stalling relies on MAPK-activated protein kinase 2 activity. Proc. Natl. Acad. Sci. USA.

[41] Wu C.C., Peterson A., Zinshteyn B., Regot S., Green R. (2020). Ribosome Collisions Trigger General Stress Responses to Regulate Cell Fate. Cell.

[42] Pedraza-Alva G., Koulnis M., Charland C., Thornton T., Clements J.L., Schlissel M.S., Rincon M. (2006). Activation of p38 MAP kinase by DNA double-strand breaks in V(D)J recombination induces a G2/M cell cycle checkpoint. EMBO J..

[43] Bulavin D.V., Amundson S.A., Fornace A.J. (2002). p38 and Chk1 kinases: Different conductors for the G(2)/M checkpoint symphony. Curr. Opin. Genet. Dev..

[44] Lemaire M., Froment C., Boutros R., Mondesert O., Nebreda A.R., Monsarrat B., Ducommun B. (2006). CDC25B phosphorylation by p38 and MK-2. Cell Cycle.

[45] Uchida S., Watanabe N., Kudo Y., Yoshioka K., Matsunaga T., Ishizaka Y., Nakagama H., Poon R.Y., Yamashita K. (2011). SCFbeta(TrCP) mediates stress-activated MAPK-induced Cdc25B degradation. J. Cell Sci..

[46] Liu K., Zheng M., Lu R., Du J., Zhao Q., Li Z., Li Y., Zhang S. (2020). The role of CDC25C in cell cycle regulation and clinical cancer therapy: A systematic review. Cancer Cell Int..

[47] Reinhardt H.C., Hasskamp P., Schmedding I., Morandell S., van Vugt M.A., Wang X., Linding R., Ong S.E., Weaver D., Carr S.A. (2010). DNA damage activates a spatially distinct late cytoplasmic cell-cycle checkpoint network controlled by MK2-mediated RNA stabilization. Mol. Cell.

[48] Lee K., Kenny A.E., Rieder C.L. (2010). P38 mitogen-activated protein kinase activity is required during mitosis for timely satisfaction of the mitotic checkpoint but not for the fidelity of chromosome segregation. Mol. Biol. Cell.

[49] Phong M.S., Van Horn R.D., Li S., Tucker-Kellogg G., Surana U., Ye X.S. (2010). p38 mitogen-activated protein kinase promotes cell survival in response to DNA damage but is not required for the G(2) DNA damage checkpoint in human cancer cells. Mol. Cell Biol..

[50] Hanahan D., Weinberg R.A. (2000). The hallmarks of cancer. Cell.

[51] Nunez G., Clarke M.F. (1994). The Bcl-2 family of proteins: Regulators of cell death and survival. Trends Cell Biol..

[52] Letai A., Bassik M.C., Walensky L.D., Sorcinelli M.D., Weiler S., Korsmeyer S.J. (2002). Distinct BH3 domains either sensitize or activate mitochondrial apoptosis, serving as prototype cancer therapeutics. Cancer Cell.

[53] Reed J.C., Cuddy M., Slabiak T., Croce C.M., Nowell P.C. (1988). Oncogenic potential of bcl-2 demonstrated by gene transfer. Nature.

[54] Yin X.M., Oltvai Z.N., Korsmeyer S.J. (1994). BH1 and BH2 domains of Bcl-2 are required for inhibition of apoptosis and heterodimerization with Bax. Nature.

[55] Kozopas K.M., Yang T., Buchan H.L., Zhou P., Craig R.W. (1993). MCL1, a gene expressed in programmed myeloid cell differentiation, has sequence similarity to BCL2. Proc. Natl. Acad. Sci. USA.

[56] Oltvai Z.N., Milliman C.L., Korsmeyer S.J. (1993). Bcl-2 heterodimerizes in vivo with a conserved homolog, Bax, that accelerates programmed cell death. Cell.

[57] Aouacheria A., Combet C., Tompa P., Hardwick J.M. (2015). Redefining the BH3 Death Domain as a ‘Short Linear Motif’. Trends Biochem. Sci..

[58] Chipuk J.E., Moldoveanu T., Llambi F., Parsons M.J., Green D.R. (2010). The BCL-2 family reunion. Mol. Cell.

[59] Lomonosova E., Chinnadurai G. (2008). BH3-only proteins in apoptosis and beyond: An overview. Oncogene.

[60] Du H., Wolf J., Schafer B., Moldoveanu T., Chipuk J.E., Kuwana T. (2011). BH3 domains other than Bim and Bid can directly activate Bax/Bak. J. Biol. Chem..

[61] Montero J., Letai A. (2018). Why do BCL-2 inhibitors work and where should we use them in the clinic?. Cell Death Differ..

[62] Whitaker R.H., Placzek W.J. (2019). Regulating the BCL2 Family to Improve Sensitivity to Microtubule Targeting Agents. Cells.

[63] Ruefli-Brasse A., Reed J.C. (2017). Therapeutics targeting Bcl-2 in hematological malignancies. Biochem. J..

[64] Crombie J., Davids M.S. (2017). Venetoclax for the treatment of patients with chronic lymphocytic leukemia. Future Oncol..

[65] Craig R.W. (2002). MCL1 provides a window on the role of the BCL2 family in cell proliferation, differentiation and tumorigenesis. Leukemia.

[66] Vairo G., Soos T.J., Upton T.M., Zalvide J., DeCaprio J.A., Ewen M.E., Koff A., Adams J.M. (2000). Bcl-2 retards cell cycle entry through p27(Kip1), pRB relative p130, and altered E2F regulation. Mol. Cell Biol..

[67] Greider C., Chattopadhyay A., Parkhurst C., Yang E. (2002). BCL-x(L) and BCL2 delay Myc-induced cell cycle entry through elevation of p27 and inhibition of G1 cyclin-dependent kinases. Oncogene.

[68] Janumyan Y.M., Sansam C.G., Chattopadhyay A., Cheng N., Soucie E.L., Penn L.Z., Andrews D., Knudson C.M., Yang E. (2003). Bcl-xL/Bcl-2 coordinately regulates apoptosis, cell cycle arrest and cell cycle entry. EMBO J..

[69] Huang D.C., O’Reilly L.A., Strasser A., Cory S. (1997). The anti-apoptosis function of Bcl-2 can be genetically separated from its inhibitory effect on cell cycle entry. EMBO J..

[70] Deng X., Gao F., May W.S. (2003). Bcl2 retards G1/S cell cycle transition by regulating intracellular ROS. Blood.

[71] Viant C., Guia S., Hennessy R.J., Rautela J., Pham K., Bernat C., Goh W., Jiao Y., Delconte R., Roger M. (2017). Cell cycle progression dictates the requirement for BCL2 in natural killer cell survival. J. Exp. Med..

[72] Whitaker R.H., Placzek W.J. (2020). MCL1 binding to the reverse BH3 motif of P18INK4C couples cell survival to cell proliferation. Cell Death Dis..

[73] Placzek W.J., Sturlese M., Wu B., Cellitti J.F., Wei J., Pellecchia M. (2011). Identification of a novel Mcl-1 protein binding motif. J. Biol. Chem..

[74] Fujise K., Zhang D., Liu J., Yeh E.T. (2000). Regulation of apoptosis and cell cycle progression by MCL1. Differential role of proliferating cell nuclear antigen. J. Biol. Chem..

[75] Harley M.E., Allan L.A., Sanderson H.S., Clarke P.R. (2010). Phosphorylation of Mcl-1 by CDK1-cyclin B1 initiates its Cdc20-dependent destruction during mitotic arrest. EMBO J..

[76] Zhong Q., Gao W., Du F., Wang X. (2005). Mule/ARF-BP1, a BH3-only E3 ubiquitin ligase, catalyzes the polyubiquitination of Mcl-1 and regulates apoptosis. Cell.

[77] Wertz I.E., Kusam S., Lam C., Okamoto T., Sandoval W., Anderson D.J., Helgason E., Ernst J.A., Eby M., Liu J. (2011). Sensitivity to antitubulin chemotherapeutics is regulated by MCL1 and FBW7. Nature.

[78] Ding Q., He X., Hsu J.M., Xia W., Chen C.T., Li L.Y., Lee D.F., Liu J.C., Zhong Q., Wang X. (2007). Degradation of Mcl-1 by beta-TrCP mediates glycogen synthase kinase 3-induced tumor suppression and chemosensitization. Mol. Cell Biol..

[79] Allan L.A., Skowyra A., Rogers K.I., Zeller D., Clarke P.R. (2018). Atypical APC/C-dependent degradation of Mcl-1 provides an apoptotic timer during mitotic arrest. EMBO J..

[80] Zhou T., Li G., Cao B., Liu L., Cheng Q., Kong H., Shan C., Huang X., Chen J., Gao N. (2013). Downregulation of Mcl-1 through inhibition of translation contributes to benzyl isothiocyanate-induced cell cycle arrest and apoptosis in human leukemia cells. Cell Death Dis..

[81] Hasan S.M., Sheen A.D., Power A.M., Langevin L.M., Xiong J., Furlong M., Day K., Schuurmans C., Opferman J.T., Vanderluit J.L. (2013). Mcl1 regulates the terminal mitosis of neural precursor cells in the mammalian brain through p27Kip1. Development.

[82] Maryanovich M., Oberkovitz G., Niv H., Vorobiyov L., Zaltsman Y., Brenner O., Lapidot T., Jung S., Gross A. (2012). The ATM-BID pathway regulates quiescence and survival of haematopoietic stem cells. Nat. Cell Biol..

[83] Maryanovich M., Zaltsman Y., Ruggiero A., Goldman A., Shachnai L., Zaidman S.L., Porat Z., Golan K., Lapidot T., Gross A. (2015). An MTCH2 pathway repressing mitochondria metabolism regulates haematopoietic stem cell fate. Nat. Commun..

[84] Wang P., Lindsay J., Owens T.W., Mularczyk E.J., Warwood S., Foster F., Streuli C.H., Brennan K., Gilmore A.P. (2014). Phosphorylation of the proapoptotic BH3-only protein bid primes mitochondria for apoptosis during mitotic arrest. Cell Rep..

[85] Kamer I., Sarig R., Zaltsman Y., Niv H., Oberkovitz G., Regev L., Haimovich G., Lerenthal Y., Marcellus R.C., Gross A. (2005). Proapoptotic BID is an ATM effector in the DNA-damage response. Cell.

[86] Zinkel S.S., Hurov K.E., Ong C., Abtahi F.M., Gross A., Korsmeyer S.J. (2005). A role for proapoptotic BID in the DNA-damage response. Cell.

[87] Hershko T., Ginsberg D. (2004). Up-regulation of Bcl-2 homology 3 (BH3)-only proteins by E2F1 mediates apoptosis. J. Biol. Chem..

[88] Brady H.J., Gil-Gomez G., Kirberg J., Berns A.J. (1996). Bax alpha perturbs T cell development and affects cell cycle entry of T cells. EMBO J..

[89] Knudson C.M., Johnson G.M., Lin Y., Korsmeyer S.J. (2001). Bax accelerates tumorigenesis in p53-deficient mice. Cancer Res..

[90] Zinkel S., Gross A., Yang E. (2006). BCL2 family in DNA damage and cell cycle control. Cell Death Differ..

[91] Ianari A., Natale T., Calo E., Ferretti E., Alesse E., Screpanti I., Haigis K., Gulino A., Lees J.A. (2009). Proapoptotic function of the retinoblastoma tumor suppressor protein. Cancer Cell.

[92] Gordon G.M., Du W. (2011). Conserved RB functions in development and tumor suppression. Protein Cell.

[93] Croxton R., Ma Y., Song L., Haura E.B., Cress W.D. (2002). Direct repression of the Mcl-1 promoter by E2F1. Oncogene.

[94] Eischen C.M., Packham G., Nip J., Fee B.E., Hiebert S.W., Zambetti G.P., Cleveland J.L. (2001). Bcl-2 is an apoptotic target suppressed by both c-Myc and E2F-1. Oncogene.

[95] Hilgendorf K.I., Leshchiner E.S., Nedelcu S., Maynard M.A., Calo E., Ianari A., Walensky L.D., Lees J.A. (2013). The retinoblastoma protein induces apoptosis directly at the mitochondria. Genes Dev..

[96] Antonucci L.A., Egger J.V., Krucher N.A. (2014). Phosphorylation of the Retinoblastoma protein (Rb) on serine-807 is required for association with Bax. Cell Cycle.

[97] Krishna M., Narang H. (2008). The complexity of mitogen-activated protein kinases (MAPKs) made simple. Cell Mol. Life Sci..

[98] Yue J., Lopez J.M. (2020). Understanding MAPK Signaling Pathways in Apoptosis. Int. J. Mol. Sci..

[99] Du L., Lyle C.S., Obey T.B., Gaarde W.A., Muir J.A., Bennett B.L., Chambers T.C. (2004). Inhibition of cell proliferation and cell cycle progression by specific inhibition of basal JNK activity: Evidence that mitotic Bcl-2 phosphorylation is JNK-independent. J. Biol. Chem..

[100] Trouillas M., Saucourt C., Duval D., Gauthereau X., Thibault C., Dembele D., Feraud O., Menager J., Rallu M., Pradier L. (2008). Bcl2, a transcriptional target of p38alpha, is critical for neuronal commitment of mouse embryonic stem cells. Cell Death Differ..

[101] Bradham C., McClay D.R. (2006). p38 MAPK in development and cancer. Cell Cycle.

[102] Kale J., Osterlund E.J., Andrews D.W. (2018). BCL-2 family proteins: Changing partners in the dance towards death. Cell Death Differ..

[103] Son J.K., Varadarajan S., Bratton S.B. (2010). TRAIL-activated stress kinases suppress apoptosis through transcriptional upregulation of MCL-1. Cell Death Differ..

[104] Azijli K., Yuvaraj S., van Roosmalen I., Flach K., Giovannetti E., Peters G.J., de Jong S., Kruyt F.A. (2013). MAPK p38 and JNK have opposing activities on TRAIL-induced apoptosis activation in NSCLC H460 cells that involves RIP1 and caspase-8 and is mediated by Mcl-1. Apoptosis.

[105] Nijhawan D., Fang M., Traer E., Zhong Q., Gao W., Du F., Wang X. (2003). Elimination of Mcl-1 is required for the initiation of apoptosis following ultraviolet irradiation. Genes Dev..

[106] Maurer U., Charvet C., Wagman A.S., Dejardin E., Green D.R. (2006). Glycogen synthase kinase-3 regulates mitochondrial outer membrane permeabilization and apoptosis by destabilization of MCL-1. Mol. Cell.

[107] Zhang K.Z., Westberg J.A., Holtta E., Andersson L.C. (1996). BCL2 regulates neural differentiation. Proc. Natl. Acad. Sci. USA.

[108] Opferman J.T., Kothari A. (2018). Anti-apoptotic BCL-2 family members in development. Cell Death Differ..

[109] Oeztuerk-Winder F., Ventura J.J. (2012). The many faces of p38 mitogen-activated protein kinase in progenitor/stem cell differentiation. Biochem. J..

[110] Min H., Ghatnekar G.S., Ghatnekar A.V., You X., Bu M., Guo X., Bu S., Shen B., Huang Q. (2012). 2-Methoxyestradiol induced Bax phosphorylation and apoptosis in human retinoblastoma cells via p38 MAPK activation. Mol. Carcinog..

[111] Cai B., Chang S.H., Becker E.B., Bonni A., Xia Z. (2006). p38 MAP kinase mediates apoptosis through phosphorylation of BimEL at Ser-65. J. Biol. Chem..

[112] Lu J., Quearry B., Harada H. (2006). p38-MAP kinase activation followed by BIM induction is essential for glucocorticoid-induced apoptosis in lymphoblastic leukemia cells. FEBS Lett..

[113] Cai B., Xia Z. (2008). p38 MAP kinase mediates arsenite-induced apoptosis through FOXO3a activation and induction of Bim transcription. Apoptosis.

[114] Sridevi P., Nhiayi M.K., Setten R.L., Wang J.Y. (2013). Persistent inhibition of ABL tyrosine kinase causes enhanced apoptotic response to TRAIL and disrupts the pro-apoptotic effect of chloroquine. PLoS ONE.

[115] Tonino S.H., van Laar J., van Oers M.H., Wang J.Y., Eldering E., Kater A.P. (2011). ROS-mediated upregulation of Noxa overcomes chemoresistance in chronic lymphocytic leukemia. Oncogene.

[116] Ambroise G., Portier A., Roders N., Arnoult D., Vazquez A. (2015). Subcellular localization of PUMA regulates its pro-apoptotic activity in Burkitt’s lymphoma B cells. Oncotarget.

[117] Chipuk J.E., Green D.R. (2009). PUMA cooperates with direct activator proteins to promote mitochondrial outer membrane permeabilization and apoptosis. Cell Cycle.

[118] Hassan M., Alaoui A., Feyen O., Mirmohammadsadegh A., Essmann F., Tannapfel A., Gulbins E., Schulze-Osthoff K., Hengge U.R. (2008). The BH3-only member Noxa causes apoptosis in melanoma cells by multiple pathways. Oncogene.

[119] Wu F., Wang Z., Gu J.H., Ge J.B., Liang Z.Q., Qin Z.H. (2013). p38(MAPK)/p53-Mediated Bax induction contributes to neurons degeneration in rotenone-induced cellular and rat models of Parkinson’s disease. Neurochem. Int..

[120] Gascon S., Murenu E., Masserdotti G., Ortega F., Russo G.L., Petrik D., Deshpande A., Heinrich C., Karow M., Robertson S.P. (2016). Identification and Successful Negotiation of a Metabolic Checkpoint in Direct Neuronal Reprogramming. Cell Stem Cell.

[121] Zhang C.L., Song F., Zhang J., Song Q.H. (2010). Hypoxia-induced Bcl-2 expression in endothelial cells via p38 MAPK pathway. Biochem. Biophys. Res. Commun..

[122] Nelyudova A., Aksenov N., Pospelov V., Pospelova T. (2007). By blocking apoptosis, Bcl-2 in p38-dependent manner promotes cell cycle arrest and accelerated senescence after DNA damage and serum withdrawal. Cell Cycle.

[123] Gupta J., del Barco Barrantes I., Igea A., Sakellariou S., Pateras I.S., Gorgoulis V.G., Nebreda A.R. (2014). Dual function of p38alpha MAPK in colon cancer: Suppression of colitis-associated tumor initiation but requirement for cancer cell survival. Cancer Cell.

[124] Scheiblecker L., Kollmann K., Sexl V. (2020). CDK4/6 and MAPK-Crosstalk as Opportunity for Cancer Treatment. Pharmaceuticals.

[125] Takenaka K., Moriguchi T., Nishida E. (1998). Activation of the protein kinase p38 in the spindle assembly checkpoint and mitotic arrest. Science.

[126] Deacon K., Mistry P., Chernoff J., Blank J.L., Patel R. (2003). p38 Mitogen-activated protein kinase mediates cell death and p21-activated kinase mediates cell survival during chemotherapeutic drug-induced mitotic arrest. Mol. Biol. Cell.

[127] Canovas B., Igea A., Sartori A.A., Gomis R.R., Paull T.T., Isoda M., Perez-Montoyo H., Serra V., Gonzalez-Suarez E., Stracker T.H. (2018). Targeting p38alpha Increases DNA Damage, Chromosome Instability, and the Anti-tumoral Response to Taxanes in Breast Cancer Cells. Cancer Cell.

[128] Gan L., Wang J., Xu H., Yang X. (2011). Resistance to docetaxel-induced apoptosis in prostate cancer cells by p38/p53/p21 signaling. Prostate.

[129] Guo X., Ma N., Wang J., Song J., Bu X., Cheng Y., Sun K., Xiong H., Jiang G., Zhang B. (2008). Increased p38-MAPK is responsible for chemotherapy resistance in human gastric cancer cells. BMC Cancer.

[130] Bragado P., Armesilla A., Silva A., Porras A. (2007). Apoptosis by cisplatin requires p53 mediated p38alpha MAPK activation through ROS generation. Apoptosis.

[131] Fang S., Qiu J., Wu Z., Bai T., Guo W. (2017). Down-regulation of UBC9 increases the sensitivity of hepatocellular carcinoma to doxorubicin. Oncotarget.

[132] Jaco I., Annibaldi A., Lalaoui N., Wilson R., Tenev T., Laurien L., Kim C., Jamal K., Wicky John S., Liccardi G. (2017). MK2 Phosphorylates RIPK1 to Prevent TNF-Induced Cell Death. Mol. Cell.

[133] Menon M.B., Gropengiesser J., Fischer J., Novikova L., Deuretzbacher A., Lafera J., Schimmeck H., Czymmeck N., Ronkina N., Kotlyarov A. (2017). p38(MAPK)/MK2-dependent phosphorylation controls cytotoxic RIPK1 signalling in inflammation and infection. Nat. Cell Biol..

[134] Dondelinger Y., Delanghe T., Rojas-Rivera D., Priem D., Delvaeye T., Bruggeman I., Van Herreweghe F., Vandenabeele P., Bertrand M.J.M. (2017). MK2 phosphorylation of RIPK1 regulates TNF-mediated cell death. Nat. Cell Biol..

[135] Puri P.L., Wu Z., Zhang P., Wood L.D., Bhakta K.S., Han J., Feramisco J.R., Karin M., Wang J.Y. (2000). Induction of terminal differentiation by constitutive activation of p38 MAP kinase in human rhabdomyosarcoma cells. Genes Dev..

[136] Haq R., Brenton J.D., Takahashi M., Finan D., Finkielsztein A., Damaraju S., Rottapel R., Zanke B. (2002). Constitutive p38HOG mitogen-activated protein kinase activation induces permanent cell cycle arrest and senescence. Cancer Res..

[137] Faust D., Schmitt C., Oesch F., Oesch-Bartlomowicz B., Schreck I., Weiss C., Dietrich C. (2012). Differential p38-dependent signalling in response to cellular stress and mitogenic stimulation in fibroblasts. Cell Commun. Signal..

